# GOLD.db: genomics of lipid-associated disorders database

**DOI:** 10.1186/1471-2164-5-93

**Published:** 2004-12-10

**Authors:** Hubert Hackl, Michael Maurer, Bernhard Mlecnik, Jürgen Hartler, Gernot Stocker, Diego Miranda-Saavedra, Zlatko Trajanoski

**Affiliations:** 1 Institute for Genomics and Bioinformatics and Christian-Doppler-Laboratory for Genomics and Bioinformatics, Graz University of Technology, Petersgasse 14, 8010 Graz, Austria

## Abstract

**Background:**

The GOLD.db (Genomics of Lipid-Associated Disorders Database) was developed to address the need for integrating disparate information on the function and properties of genes and their products that are particularly relevant to the biology, diagnosis management, treatment, and prevention of lipid-associated disorders.

**Description:**

The GOLD.db  provides a reference for pathways and information about the relevant genes and proteins in an efficiently organized way. The main focus was to provide biological pathways with image maps and visual pathway information for lipid metabolism and obesity-related research. This database provides also the possibility to map gene expression data individually to each pathway. Gene expression at different experimental conditions can be viewed sequentially in context of the pathway. Related large scale gene expression data sets were provided and can be searched for specific genes to integrate information regarding their expression levels in different studies and conditions. Analytic and data mining tools, reagents, protocols, references, and links to relevant genomic resources were included in the database. Finally, the usability of the database was demonstrated using an example about the regulation of Pten mRNA during adipocyte differentiation in the context of relevant pathways.

**Conclusions:**

The GOLD.db will be a valuable tool that allow researchers to efficiently analyze patterns of gene expression and to display them in a variety of useful and informative ways, allowing outside researchers to perform queries pertaining to gene expression results in the context of biological processes and pathways.

## Background

The excessive consumption of high calorie, high fat diets and the adoption of a sedentary life style have made obesity and atherosclerosis major health problems in Western societies. In the USA, over 50% of the population are over-weight (BMI > 25) and close to 25% are considered obese (BMI > 30) [1,2]. As a consequence, a large fraction of the population is at risk to develop a broad range of common, life-threatening diseases including non-insulin dependent diabetes, various hyperlipidemias, high blood pressure and atherosclerosis. Vascular disease including coronary heart disease and stroke is currently the major cause of death in the United States and in other industrialized nations.

At the root of obesity and atherosclerosis is an excessive deposition of neutral lipids. Adipose tissue accumulates predominantly triglycerides, whereas macrophages along the blood vessel wall mainly accumulate cholesterol and cholesteryl esters. Accordingly, a detailed understanding of the molecular mechanisms that govern the balance between lipid deposition and mobilization is fundamentally important for the prevention and improved treatment of disease. In addition to the apparent environmental components involved in the pathogenesis of disorders related to lipid and energy metabolism, a large number of studies have provided undisputed evidence that susceptibility genes contribute around 50% of the phenotype. These genes encode products involved in the cellular uptake, synthesis, deposition and/or mobilization of lipids. However, characterization of many if not most of these genes and their products remains rudimentary. Deficiencies in the current level of understanding extend to key enzymes such as important triglyceride hydrolases in adipose tissue [3] or cholesteryl ester hydrolases in macrophages, hormones, signal transduction pathways, and the regulation of the transcription of relevant genes.

While medical molecular biology traditionally associates single genes and gene products with diseases, a growing body of evidence suggests that several common disease phenotypes arise from the delicate interaction of many genes as well as gene-environment interactions. To elucidate the development of obesity and atherosclerosis, it will be necessary to analyze patterns of gene expression and relate them to various metabolic states. To discover novel genes, processes and pathways that regulate lipid deposition and mobilization, a departure from hypothesis-driven research and turn to a discovery-driven approach is necessary. The application of high-throughput technologies and genome-based analysis will provide the tools for the analysis of gene-gene and gene-environment interactions in a systematic and comprehensive manner.

To facilitate genomic research we have initiated the development of a system for storing, integrating, and analyzing relevant data needed to decipher the molecular anatomy of lipid associated disorders. In order to provide a reference for pathways and information of the relevant genes and proteins in an efficiently organized way, we have created the Genomics Of Lipid-Associated Disorders database (GOLD.db). The GOLD.db integrates disparate information on the function and properties of genes and their protein products that are particularly relevant to the biology, diagnosis management, treatment, and prevention of lipid-associated disorders.

## Construction and content

The main goal of the GOLD.db was to provide biological pathways with image maps and visual pathway information. For each element in the pathway, specific information exists including structured information about a gene, protein, function, literature, and links. The GOLD.db provides also the possibility to map gene expression data individually to each pathway. Additionally, analytic and data mining tools, reagents, protocols, references, and links to relevant genomic resources were included in the database.

The GOLD.db was implemented in Java technology [4]. Hence, the pathway editor, as well the web application are platform independent. The web application of GOLD.db is build in Java Servlets and JavaServer Pages technology based on the Model-View-Controller Architecture [5]. For the implementation, the freely available struts framework [6] was used. This code can be easily deployed in any Servlet Container. We used the Servlet Container Tomcat (also freely available at [7]) which is accessible from all web browsers. Oracle 9i was used as database management system. The interface between the Java and the Database management system was established using Java database connectivity (JDBC) 2.0. Therefore, migration to other freely available DBMSs like mySQL can be easily done. For additional storage and communication between the pathway-editor components, the markup language XML containing structured, human readable information, was used. The provided pathways can be downloaded as Scalable Vector Graphics (SVG) [8], a standard for describing two-dimensional graphics in XML, and can be visualized in this format on the client side with the web browser using a plug-in for SVG.

For tracking the repository of the reagents like clone resources and libraries which can be used for microarray studies, we have developed a relational database. Information about the vector, the sequence and length of the clone insert, primers for the PCR amplification, tissue, organism, accession number, library, container, storage information, date and person and access to other clone bases (e.g. IMAGE Consortium) can be stored. Users of the GOLD.db can list these clones and get all the information about each available clone. With restricted access, clone information or even clone lists can be uploaded and selection lists can be created and deleted. The input mask is designed in such way that the user can choose one of the elements of the created selection lists.

In order to deal with the huge amount of data associated with large scale studies and to perform sequence based and microarray analysis, several bioinformatic tools were integrated or can be downloaded. Sequence similarity search against databases can be performed with BLAST (Basic Local Alignment Search Tool) [9], FASTA [10] or HMM (Hiden Markov Models) [11] on a 50 CPU Myrinet Cluster. The sequence retrieval system SRS (LION Bioscience AG, Heidelberg, Germany) was included to enable rapid, easy and user friendly access to the large volumes of diverse and heterogeneous data [12]. The latest version of the PathwayEditor for the construction of biological pathway diagrams can be downloaded. For microarray analysis the platform independent JAVA tools ArrayNorm [13] for normalization of microarray data and Genesis [14] for clustering and analysis of large scale gene expression datasets were made available.

## Utility and discussion

### Pathways

In order to construct the biological pathways of interest, we have developed a pathway editor [15] and an extended version to map gene expression data (pathway mapper). This drawing tool provides the possibility to draw elements – typically representing a gene as part of the pathway – and the connection between those elements. The benefit of this tool is that information can be appended to each element via an input mask. This information can be accessed by clicking on the corresponding element in the image map within the pathway mapper or when saved and uploaded via the web interface to the GOLD.db. To design this pathway service as flexible as possible, features are provided for the remove, up- and download of relevant pathways (image maps) including the underlying additional information of the elements. However, this service is on a restricted basis to prohibit unauthorized access. Since some pathways tend to become very detailed an option to search for genes or gene accession number, respectively, within the pathways was built in. The pathway editor is executable as a standalone application and is available from [16]. Currently annotated pathways are the insulin signaling pathway, the IGF-1 pathway and the adipogenesis regulatory network. Other pathways of lipid metabolism will follow in the near future. Available KEGG pathways can also be adapted with the pathway editor based on the provided XML files [17] and uploaded in the same way. All relevant KEGG pathways for different organisms are provided. Moreover, pathways from BioCarta were made available within the GOLD.db and HTML files [18] were parsed to provide additional meta-information of the pathway elements.

For each element in the pathways a specific information field exists. The field includes structured information about a gene, protein, function, literature, and links to well-curated and annotated databases. Besides the gene name and the symbol name – for human the HUGO symbols and gene names and for mouse the MGI nomenclature were used – RefSeq numbers for the transcript and the protein as well as a link to SwissProt/UniProt and LocusLink is available. For the elements of the KEGG pathways a link to the provided enzyme or product information was given. The description, localization and classification of the factors are entered by the annotator in plain text and are accessed in the same format. The references used to generate the content of the database entries can be appended, including a link to the PubMed entry. There is also the possibility to create a list of reference entries for the pathway. If a clone for a specific gene is available in the clone resources, the clone name will be displayed automatically and a link with optional information about this clone is provided.

### Mapping of gene expression data sets to pathways

Through the integration of several types of biological information deeper insights into the molecular mechanisms and biological processes can be gained than just by the analysis of one type of experimental results. In the GOLD.db it is possible to map gene expression data (for instance results of microarray studies) to the corresponding elements of the available pathways similar to previous efforts [19]. Either an individual or a provided gene expression data set can be used to visualize the gene expression at different experimental conditions sequentially or all at once in the context of a pathway. If an element (gene) of the pathway is included in the data set, the related symbol in the image map is color coded according to the relative gene expression or the log ratio in two color microarray experiments, respectively.

As key for the mapped relation the RefSeq number [20] is used. Hence, only those elements in the data set file are mapped, where the RefSeq number in the data set is specified. For the KEGG pathways each element classified by the enzyme classification number (EC) is virtually subdivided into different corresponding RefSeq entries, since one EC is represented by one or more RefSeq entries.

### Curated gene expression data sets

Analysis of gene expression patterns in animal and cell models for lipid-associated disorders will help to understand the fundamental gene relations and regulatory mechanisms responsible for the development of obesity related diseases. The huge amount of data associated with the analysis of large scale gene expression analysis raises the demand of tools for storing, processing and retrieving complex information. Although a number of studies have been published and despite the requirements of some journals to deposit microarray data in public databases like GEO  or ArrayExpress , it is still very difficult for researchers to obtain the original data. Web sites with Supplementary information are not maintained and/or not further developed. Hence, a database with a large collection of curated datasets will be enormously valuable for the community. Approaches to upload and retrieve gene expression data were pursued within the GOLD.db. Large scale gene expression data sets can be uploaded in form of tab delimited text files (Stanford file format) [21] as used for cluster analysis programs together with additional information about the experimental conditions and the citation for already published data sets. Within those data sets the search for specific genes is possible to provide integrated visualization of gene expression levels in different studies and experimental conditions.

### Example for using GOLD.db: regulation of Pten during adipocyte differentiation

**Figure 1:**
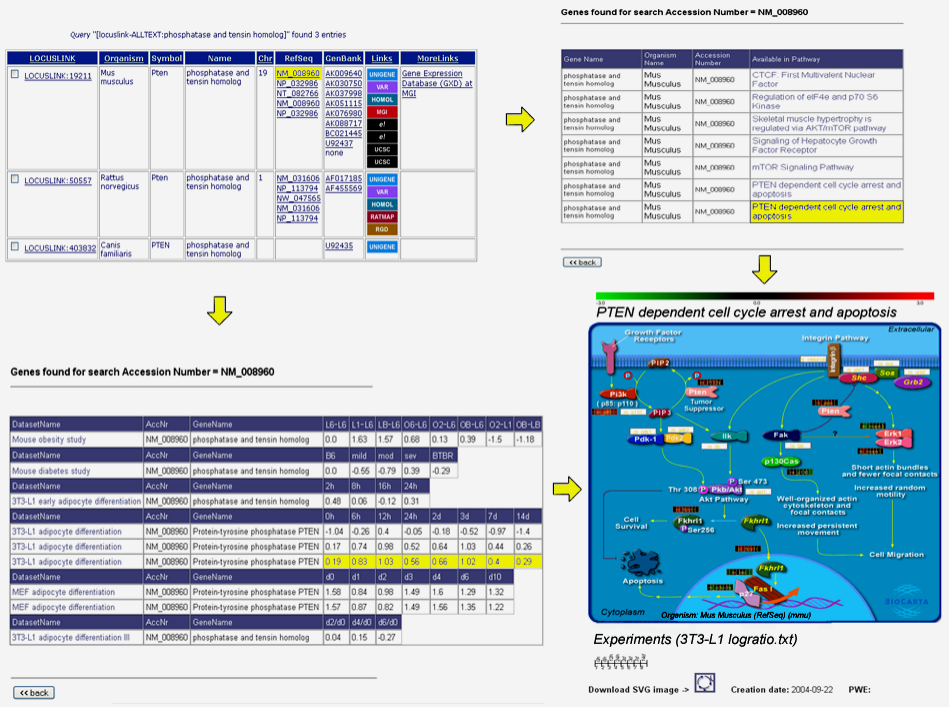
Various result tables from using GOLD.db to address the question how is PTEN regulated during adipocyte differentiation (*top left: *result of search in SRS for phosphatase and tensin homolog; *top right: *pathways, in which PTEN is involved; *bottom left: *relative gene expression levels of PTEN in different datasets; *bottom right: *PTEN dependent cell cyle pathway with mapped gene expression levels)

Recently, it was shown that insulin sensitivity, energy expenditure, and thermogenesis were enhanced in adipose-specific Pten-deficient (AdipoPten-KO) mice. Body and adipose tissues weight in these mice were significantly lower than those of control mice in spite of a larger food intake [22]. We addressed the question how is the expression of the Pten gene regulated during adipocyte differentiation in different models and experimental setups and in which pathways is PTEN involved. The workflow for the analysis is described in Figure 1. Pten (phosphatase and tensin homolog deleted on chromosome 10) is known as tumor suppressor gene and is a protein and lipid phosphatase with the major substrate phosphatiylinositol 3,4,5-triphosphate (PIP3), as indicated in the annotated insulin signaling pathway within the GOLD.db. In fact, Pten regulates negatively the insulin signaling pathway in 3T3-L1 adipocytes [23].

During adipocyte differentiation cyclin dependent kinase inhibitors, like p21 leads to a hypophosphorylation of the Retinoblastoma protein (Rb) which allows binding to the E2F transcription factor, causing cells to permanently exit the cell cycle – a required step in adipocyte differentiation called mitotic clonal expansion – before entering the terminal differentiation state. pRb interacts physically with adipogenic CCAAT/enhancer-binding proteins and positively regulates transactivation by C/EBPβ and therefore plays a pivotal role in adipocyte differentiation [24,25]. Hence, since a) PTEN is expressed during adipogenesis (Figure 1), b) is involved in the regulation of Rb [22], a major player in adipogenesis, and c) is an important component in cell cycle arrest and apoptosis (Figure 1), it can be postulated that PTEN plays an important role in fat cell development.

Thus, using recently identified key player for food intake and weight control and using the GOLD.db, it is possible to address relevant questions and generate testable hypotheses on the molecular mechanisms of fat cell development.

## Conclusions

The vast quantity of gene expression data generated in genomic studies presents a number of challenges for their effective analysis and interpretation. In order to fully understand the changes in expression that will be observed, we must correlate these data with phenotype, genotype, metabolism and other information including the tissue distribution and time course expression data gleaned from previous studies. The goal of our work was the development of a specialized database and tools that allow researchers to efficiently analyze patterns of gene expression and to display them in a variety of useful and informative ways, allowing outside researchers to perform queries pertaining to gene expression results in the context of biological processes and pathways. The uniqueness of the GOLDdb database we have developed is threefold: 1) the inclusion of annotated pathways, 2) the availability of curated datasets and 3) the possibility to map experimental data on biological pathways. The upcoming challenges will be to include data from functional analysis and proteomics data, which will give us new opportunities in understanding mechanisms of different applications and lipid-associated disorders in particular.

## Availability and requirements

The GOLD.db database should be cited with the present publication as a reference. Access to GOLD.db is possible through the world wide web at . The pathway editor and the clone tracker are available free of charge to academic, government, and other nonprofit institutions.

## Author's contributions

HH was responsible for the content, the annotation process, webdesign, and processing of data sets. MM was responsible for the implementation of the database and web application as well as the relational database for the clone tracker. BM and JH had implemented the mapping of expression data to pathways. GS is involved in providing of sequence analysis tools and server software. DMS has annotated the insulin signaling pathway. ZT was responsible for the design of the study and for overall project coordination.

## References

[B1] Flegal KM, Carroll MD, Kuczmarski RJ, Johnson CL (1998). Overweight and obesity in the United States: prevalence and trends, 1960-1994. Int J Obes Relat Metab Disord.

[B2] Must A, Spadano J, Coakley EH, Field AE, Colditz G, Dietz WH (1999). The disease burden associated with overweight and obesity. JAMA.

[B3] Zechner R, Strauss J, Frank S, Wagner E, Hofmann W, Kratky D, Hiden M, Levak-Frank S (2000). The role of lipoprotein lipase in adipose tissue development and metabolism. Int J Obes Relat Metab Disord.

[B4] (2004). Java Technology. http://java.sun.com.

[B5] Gamma E, Helm R, Johnson R, Vlissides J (1995). Design Patterns - Elements of Reusable Object-Oriented Software.

[B6] (2004). Struts framework. http://jakarta.apache.org/struts/.

[B7] (2004). Tomcat. http://jakarta.apache.org/tomcat/.

[B8] (2004). SVG. http://www.w3.org/TR/SVG.

[B9] Altschul SF, Gish W, Miller W, Myers EW, Lipman DJ (1990). Basic local alignment search tool. J Mol Biol.

[B10] Pearson WR, Lipman DJ (1988). Improved tools for biological sequence comparison. Proc Natl Acad Sci U S A.

[B11] Eddy SR (1998). Profile hidden Markov models. Bioinformatics.

[B12] Etzold T, Ulyanov A, Argos P (1996). SRS: information retrieval system for molecular biology data banks. Methods Enzymol.

[B13] Pieler R, Sanchez-Cabo F, Hackl H, Thallinger GG, Trajanoski Z (2004). ArrayNorm: comprehensive normalization and analysis of microarray data. Bioinformatics.

[B14] Sturn A, Quackenbush J, Trajanoski Z (2002). Genesis: cluster analysis of microarray data. Bioinformatics.

[B15] Trost E, Hackl H, Maurer M, Trajanoski Z (2003). Java editor for biological pathways. Bioinformatics.

[B16] (2004). Institute for Genomics and Bioinformatics, Graz University of Technology. http://genome.tugraz.at.

[B17] Kanehisa M, Goto S, Kawashima S, Nakaya A (2002). The KEGG databases at GenomeNet. Nucleic Acids Res.

[B18] (2004). Biocarta Pathways. http://biocarta.com/genes/allPathways.asp.

[B19] Dahlquist KD, Salomonis N, Vranizan K, Lawlor SC, Conklin BR (2002). GenMAPP, a new tool for viewing and analyzing microarray data on biological pathways. Nat Genet.

[B20] Pruitt KD, Maglott DR (2001). RefSeq and LocusLink: NCBI gene-centerd resources. Nucleic Acids Res.

[B21] Eisen MB, Spellman PT, Brown PO, Botstein D (1998). Cluster analysis and display of genome-wide expression patterns. Proc Natl Acad Sci U S A.

[B22] Komazawa N, Matsuda M, Kondoh G, Mizunoya W, Iwaki M, Takagi T, Sumikawa Y, Inoue K, Suzuki A, Mak TW, Nakano T, Fushiki T, Takeda J, Shimomura I (2004). Enhanced insulin sensitivity, energy expenditure and thermogenesis in adipose-specific Pten suppression in mice
2. Nat Med.

[B23] Nakashima N, Sharma PM, Imamura T, Bookstein R, Olefsky JM (2000). The tumor suppressor PTEN negatively regulates insulin signaling in 3T3-L1 adipocytes. J Biol Chem.

[B24] Hansen JB, Petersen RK, Larsen BM, Bartkova J, Alsner J, Kristiansen K (1999). Activation of peroxisome proliferator-activated receptor gamma bypasses the function of the retinoblastoma protein in adipocyte differentiation. J Biol Chem.

[B25] Chen PL, Riley DJ, Chen Y, Lee WH (1996). Retinoblastoma protein positively regulates terminal adipocyte differentiation through direct interaction with C/EBPs. Genes Dev.

